# Medical and Philosophical Intelligence

**Published:** 1811-11

**Authors:** 


### MEDICAL AND PHILOSOPHICAL INTELLIGENCE.

*Extract from the Report of the Vaccine Pock Institution in Broad-Street.*

"1. With regard to the occurrence of small-pox subsequent to vaccination—out of 5000 patients registered in our tables, it appears that nine have subsequently taken the small-pox, as admitted by the Medical Establishment:—they had all gone through the distinct stages of vaccination:—the matter with which some of them had been inoculated was from patients ascertained to have been rendered unsusceptible of the small-pox; and some of them had furnished matter for inoculation which rendered persons incapable of taking the small-pox, as appears by the test of variolous inoculation. Some of them had evidently constitutional disorder at the usual time. They had all distinct scars. Four of these failures occurred in two families, viz. two in Maber's and two in Lemon's. None had dangerous symptoms, and the pocks scabbed sooner than usual in most of them; sooner by at least two or three days; most of them had the disease so mildly that they would have been considered as even the mildest cases of inoculated small-pox. These failures induced the Medical Establishment to inquire concerning their occurrence in the practice of others: a great number of communications were accordingly made; but after investigation, not more than forty* were found to be substantiated: of these, three died. Most of them had the small-pox very mildly; more so than the inocu-

---

* Three more failures have been communicated since this paper was written, viz. One the son of a person of high rank, by Mr. Walker, at present the general topic of conversation; the other, by Mr. Tegart; the third, a very decisive one, was communicated by Mr. Marshall.

lated lated small-pox; some having merely a slight fever, and an eruption of pimples, or of vesicles, so small, and so speedily scabbed, that they would not perhaps have been allowed to be the variolous disorder, if other children in the same family had not been ill in the distinct sort, and who had apparently infected one another. Two of these failures occurred on inoculation for the small-pox; and the rest, of course, occurred in the natural way.

"2. It has been proved that a large proportion of our patients have been rendered secure against the small-pox by means of re-inoculation of above sixty children publicly, in 1804 *, at the Small-Pox Hospital; and of several hundreds, at different times, at the Institution, with small-pox matter.

"3. A great number of our patients, it is well known, have been repeatedly exposed to the effluvia, or contact, with persons in the small-pox; and hence such a degree of confidence has been produced, that notwithstanding the proposed reward of five guineas to any who should take the small-pox after having been certified to be secure by the medical officers, very few would submit to the test required of the second inoculation.

"4. One of the physicians of this Institution, (Dr. Pearson), after repeated trials, found that a person who had gone duly through the cow-pox, was equally incapable of being infected with the vaccine matter a second time, as by the variolous. Many trials of this kind were instituted publicly, at the Institution, in 1807, in the presence of several Fellows of the College, to shew that the re-inoculation with vaccine matter was equally a test of security of the constitution against the small-pox as variolous matter. The same member announced the result of his experience on this point as early as 1799. In the printed directions of the Institution for Vaccination, in January, 1801, the test of re-inoculation is recommended, by the Institution ‡, to answer the same purpose as inoculation with variolous matter. To afford the most decisive proof, vaccine matter has been often inserted into one arm and variolous into the other, or both in different parts of the same arm; and in no instance has the small-pox been produced at all, or the vaccine a second time: but if a local affection, viz., a large pimple, or a small vesicle, has been produced by one kind of matter, it has been equally produced by the other; and if no affection at all was excited by one, there was none by the other.

"5. Numerous trials have shewn, at the Institution, that the same constitution cannot take the cow-pox subsequently to the small-pox, contrary to a high authority, who asserts also, that the cow-pox is producible a second time. Hence the variolous matter and the vaccine are mutually tests, or afford a counter-proof, of each other having affected the human subject, to be incapable of taking either of these diseases a second time.

---

* See Statement of Evidence, 1804.

‡ See the Minute Books.

"6. We

"6. We have made a great many trials, by which, perhaps, may be determined the exact period when the constitution is by vaccination rendered incapable of being affected by the small-pox, and which appear in our minute-book, viz. by re-inserting either vaccine or variolous matter on any one or each succeeding day from the first insertions. It has appeared that the succeeding insertions can only produce pimples, or vesicles, of the figure and magnitude they usually do, according to the time after inoculation, but cease to grow larger at the period the constitution is supposed to be affected by the first inoculation. Mr. Bryce has proposed these re-insertions, during the vaccination, as a test; and we agree with him that they may be relied upon; but we do not think they will be so satisfactory to the patients as the second inoculation, some weeks, or longer, after the first.

These experiments, if the explanation could be given in this place, would prove further the fact ascertained, that a person who has gone through the small-pox cannot take the cow-pock, nor can a person who has gone through the cow-pock take it a second time.

"7. Farther proofs of the proposition just stated may be found in the trials at the Institution, of inserting variolous and vaccine matter in different places of the same arm on the same day, in which cases the respective vesicular pocks of each kind of matter are produced in the inoculated parts; but in some cases the small-pox fever and eruption are produced, and in others only the vaccine affection.

"8. The inoculation, with a mixture of the vaccine and variolous matter, does not produce a hybrid disease, but sometimes the cow-pock distinctly, and at other times the small-pox only.

"9. With respect to the subsequent health: children have been frequently brought to the Institution some time after the cow-pock, with various eruptive complaints; but as most or all of these have been seen in those who have not been vaccinated, we doubt whether they can be reasonably imputed to vaccination. If any of these be peculiarly the consequence of inoculation, it is a kind of rash not unlike the red gum, and also the tooth rash, the nettle rash, and some undescribed cutaneous affections.

"10. We have only seen one great anomaly in the course of our practice, which occurred four years ago, in August and September. The vaccine pocks had not their usual distinct figure; a cutaneous affection attended; and the patients had sore arms; but they appeared to be quite unsusceptible of the small-pox, and in a few months the same matter, by succession, produced the regular vaccina.

"11. We have had very little trouble with sore and inflamed arms, especially the last three or four years.

"12. It is now twelve years since the original matter for the Institution was taken from the cows by Dr. Pearson, and we can perceive no deterioration or difference in its effects.

"13. With respect to the effects of matter, according to the state of the pock, of the arm, and the constitution, the result of eleven years experience is—

"1. That

"1. That matter taken before the scabbing begins, is the most efficacious.

"2. If taken later, there is no other consequence but frequent failure to infect.

"3. When the scab itself was used, it in no instance produced the cow-pock.

"4. The presence or the absence of the areola appeared to be of no consequence.

"5. No difference has been observed according to the health of the patient from whom it is taken.

"6. The rule for taking the matter should be according to the state of the pock, and not according to the day after inoculation; but the usual course is such, that in general it is more efficacious before the 8th or 9th day than later.

"7. Matter of the usual, regular, distinct pock, should always be preferred: not perhaps on any other account but to prevent disappointment in producing the vaccine affection.

"8. We have met with no such matter as that called *spurious*, or which produces a similar affection somewhat like the cow-pock by inoculation from subject to subject, but does not destroy the susceptibility of the small-pox.

"14. The deviations from the usual course are imputable

"1st. To certain peculiarities of the constitution,

"2dly. To the pre-occupancy and intervention of other diseases, and

"3dly. To cutaneous affection of the part inoculated.

"15. We have not been able to observe a constitutional disorder on the fourth day referable to the agency of the matter on the constitution, distinct from that usually on the ninth day from the local affection.

"16. The inoculation of vaccine matter, in several places in each arm, does not appear to produce more local inflammation than a single pock, but it seems attended with the advantage of more frequently affecting duly the constitution.

"17. In a few instances the cow-pock cannot be produced at all even by repeated inoculation; and in such cases the small-pox cannot be produced; there being, perhaps, a connate unsusceptibility of both disorders.

"18. We have seen no unusal inflammation from repeatedly inserting matter of small-pox, or cow-pock, to determine the question of susceptibility of either of these disorders.

"19. No danger is to be apprehended from the inoculation for the cow-pock at any time after exposure to the small-pox: the two diseases do not subsist together constitutionally; so that nothing can be lost, and advantage may be gained by vaccination.

"20. We have had no deaths by vaccination, as already said, in our own practice; but accounts have been communicated—1st. Of the case of an infant of which we could get no particulars to determine the cause of the death;—2d. A few cases proved fatal in the practice of others, from the state of the arms;—3d. By deficiency of of food, by exposure to cold, and externally injuring the arms inoculated.

"21. With regard to the influence of vaccination in diminishing the mortality by small-pox, the following statement may give some satisfaction, or serve to assist the judgment. For this purpose we shall state the number of deaths by small-pox from the bills of mortality of parish clerks, of London, during the twelve years since vaccination was introduced, viz. from January 1799 to January 1st, 1811; and also during the twelve years immediately preceding the vaccine practice, viz. from the 1st of January, 1787, to January the 1st, 1799. But in order to judge more accurately, we shall arrange the two periods of twelve years under three heads, each comprehending four years."

*(To be concluded in the next Number.)*

---

INSTITUTE OF FRANCE.—M. Odier, of Geneva, in a memorial read before the first class of the Institute, on the 18th of February, attempts to prove the advantages that would be derived from a permanent endowment, for the purpose of sending physicians to visit foreign universities. "This project," he observes, "has been realized in England by Dr. Radcliff, who proposed that two graduates of the University of Oxford should receive a certain revenue for their support during six years, on condition of passing five years of that time out of Great Britain." The little care in making a proper choice of these physicians, and the want of regulations to exact from them an account of the employment of their time, have paralysed an institution which, M. Odier thinks, might prove essentially advantageous. He has drawn up a table of measures which should be pursued to derive the utmost possible benefit from the plan, and render it worthy of Imperial munificence.

The travelling physicians should visit foreign schools; should inform themselves of the therapeutics, and mode of instruction there taught; should themselves observe on the spot, the characters of endemics; the shades of difference caused in epidemic and sporadic diseases by climate and habit; should especially extend the catalogue of Materia Medica, either by introducing into general practice remedies of which the use is confined to a particular country, or by making known properties in a medicament different from those which we have assigned to it; finally, they should carefully note the police of hospitals, and the modes of practice therein pursued.

Without pretending to refute the arguments of M. Odier, or to attack the supposed advantages of his project, we may remark, that Medical Journals are sufficiently numerous to maintain a communication between all the practitioners of the civilized world; offering an effectual means of publishing and readily circulating opinions, facts, and observations, which could not properly form a subject of a special work; or which, if so published, would not, in so short a period of time, obtain the same degree of publicity.

We may also observe that, unless these travelling physicians possessed a sound judgment, (which is rare) they would return from their tours, fraught with the errors, and useless and trifling productions of the countries they visited, without obtaining any new information, or collecting any thing valuable or essentially important, whilst no discovery of consequence sequence remains long unpublished. When do we hear of any interesting work, or see any brilliant speculation realized by those who, after having been long Cosmopolitan physicians, have ultimately fixed their residence at Paris?

As for the penury of the Materia Medica, which, according to M. Odier, demands the adoption of his projects as a means of enlarging that branch of science, by importing new treasures, we think, that so far from being limited, it is much too extended. For, though many learned physicians have laboured to simplify it, this science still embraces a variety of useless articles. To prove the acquisitions which might be made to the Materia Medica, M. Odier cites the use which he has made of the *muriate suroxigéné de potasse.* This salt, given in a case of anasarca for which a variety of means had proved ineffectual, acted as a powerful diuretic; "every thing seemed to be going on well, when unfortunately," says M. Odier, "the patient died suddenly, *le malade mourut subitement.* (*Journal de Med.*) J. B. N.

---

The third session of the medico-chirurgical society of the University of New York was holden during the winter of 1809 and 1810. The following are the titles of the essays read at the sittings of the society.

1. Upon Typhus; 2. Upon the vitality of the blood; 3. Upon Nicotiana tabacum; 4. Upon hepatitis; 5. Upon Conception; 6. Upon pregnancy as a state of disease; 7. Upon respiration; 8. Upon Diabetes; 9. Upon life; 10. Upon the existence of frigorific particles; 11. Upon mania; 12. Upon Generation in different animals; 13. Upon the theories of generation; 14. Upon the febrile diseases of Orange county, and the treatment which proved most successful; 15. Upon dysentery; 16. Upon *febris introversa*; 17. Upon parturition; 18. Upon spasm; 19. Upon phthisis pulmonalis; 20. Upon the cause of inflammation; 21. Upon anasarca; 22. Upon acute rheumatism; 23. Upon croup; 24. Upon the non-existence of sympathy; 25. Upon animal heat; 26. Upon the nature, cause, and treatment of yellow fever in the United States and West Indies; 27. Upon fevers; 28. Upon cynanche trachealis; 29. Upon theoretic errors; 30. Upon the sedative properties of opium; 31. Upon the nyctalopia which has appeared in the prison of New York; 32. Upon dysentery; 33. Upon necrosis; 34. Upon the nourishment of the fœtus in utero; 35. Upon the treatment of dysentery; 36. Upon the mode of communication between the mother and fœtus.

Attention is beginning to be paid to medical topography in some states of the American federation. It has been discovered that goitre has become more common in certain countries to the west of the States. Epidemic catarrhs, during the last three years, have been the subject of several memoirs. The diseases of domestic animals, especially those affections common to the human species and some tribes of the brute creation, have fixed the attention of naturalists. Experiments and observations upon cutaneous absorption continue, particularly in Philadelphia, where a difference of opinion respecting that function is maintained. Chemistry, botany, and natural history are cultivated in all the great cities with extraordinary zeal.

Professor B. S. Barton, a learned naturalist of Philadelphia, has composed posed two memoirs on the *Opossum*, an animal peculiar to North America, of the species *didelphis marsupialis*; but he names it *didelphis wooapink*; it is *le sarique de Buffon*. The female of this species has a bag or pouch beneath the belly. In the first of these memoirs, the author gives at length the natural history of the animal; indicates the place which it ought to occupy in the system, its nourishment, habits, the places on the continent where it may be found, the periods of coupling, &c. He describes in the female, the whole process of utero-gestation, which comprehends a period of 22 to 26 days.

The second memoir treats of the second epoch of gestation, which the author terms marsupial gestation; it dates from the moment when the embryos pass from the uterus into the pouch, and is longer than the uterine gestation. The Professor has ascertained the volume and the weight of several embryos immediately after their exclusion from the uterus. One of them weighed only a grain; six others weighed rather more. These observations have been confirmed by M. Palisot de Beauvois, member of the Institute.

The mechanism by which the embryos quit the uterus, glide to the teats, and adhere to them by an invariable and determined instinct, is regarded as one of the most astonishing phenomena which natural history presents. They remain in their new situation about fifty days, in which time they have attained the size of a common mouse; they then detach themselves from the teats, but return to them till they have reached the size of a rat, when they entirely leave the pouch and feed on flesh and vegetables.

It has been supposed, and especially by Vic D'Azyr, that the mother assists the abortion with her paws, and places the embryos in the pouch. Professor Barton proves that this opinion is erroneous. He also disproves the assertion of Beverly (in the History of Virginia) and many others in the United States, who pretend that generation is effected in the false belly, where the embryos grow, attached to the teats. He has proved that young opossums, weighing nine grains each, cannot be detached from the pouch, which is as a second uterus, without some loss of blood.

---

Dr. Kiesser, of Nordheim, has given an account in Hufeland's Journal of a diuretic linament which he considers as specific in retention of urine from spasm. It is composed of half an ounce of oil of turpentine, two drachms of the yolk of fresh eggs; these are to be rubbed in a glass or earthen-ware mortar till perfectly mixed, when six ounces of peppermint water should be added. The liniment is to be applied to the inguinal region. After some frictions, in general, the violent spasm ceases and the urine flows readily.

In the same Journal, a singular fact is related by Consbruch. He knew a family in which the male children were subject from their youth to fatal hæmorhages, which took place spontaneously, either from the nose, or from the slightest wounds. The hæmorrhage sometimes could not be stopped by any means, and two individuals sunk under it in spite of every remedy. The females, as well as the parents, were entirely exempt from this peculiarity; but two male infants of one of the daughters in this family were subject to it; one of them died in consequence of hæmorrhage occurring from a very slight wound; the other is still living, living, but is subject to hæmorrhage from the nose in spring and autumn; when the hæmorrhage does not occur, he is subject to severe and continued attacks of gout. To avoid these, it is necessary to bleed him from the arm at least once in the spring. The blood, in this case, cannot be stopped by common means; strong compression is obliged to be used for several weeks. The uncle of this young man, who, in his youth, had been subject to alarming hæmorrhages; is not always exempt from a return; he also suffers much from the gout. Towards the end of the paroxysms, considerable echymosis appears on the parts which have chiefly been affected with gout. The other individuals of this family enjoy perfect health. The subjects of the hæmorrhage are distinguished by dark and sparkling eyes, black hair, and the complexion of the atrabilious temperament.

---

We have seen, within these few days, another case of small-pox, occurring some years after the person had gone through the casual disease in its severest form. This person, now 35 years of age, had confluent small-pox in her youth, (sufficient evidence of which now remains on her face), about a month since, nursing her child in *Variola confluenta*, received the disease. The eruption, which appeared only where the child came in contact with the skin of the mother, was preceded by the usual symptoms of fever. On one side of the face, where it appears that the face of the child was laid against the mother's, numerous pustules, large, distinct, and going through the regular stages, were placed. On the nipples, where the child had applied its lips, and on the areola, pustules also appeared. A few pustules were scattered on the mother's arms where the naked surface had been placed in contact, with the eruption on the child. The mother had likewise pustules in her mouth, where the spoon with which she fed the child had touched. This case presents a curious anomaly in the history of *Variola*. It appears, from the statement given by the woman, that she was ill three or four days before the eruption shewed itself; but if this fever was the genuine eruptive fever of Variola, it is obvious that the susceptibility in the system had been so modified by the patient having before passed through the disease, that it was incapable of producing pustules but where the variolous fluid was applied by actual contact.

---

The Lectures which are annually given at the *Scientific Institution*, in *Princes Street, Cavendish Square*, will commence on the 20th of Nov. and will consist of a popular course of twelve Lectures on the most interesting branches of experimental science:—twelve Lectures on chemistry, comprising an exposition of all the recent discoveries; by *Mr. George Singer*:—and twelve Lectures on the philosophy of the mechanic arts, with new experiments on the tenacity of metals; by *Mr. E. Lydiatt*. These Lectures are delivered every Tuesday and Saturday, at eight o'clock in the evening.

---

Mr. Pearson, F. R. S. Senior Surgeon of the Lock Hospital, &c. is now engaged in delivering a course of Lectures on syphilis, cachexia syphiloidea, gonorrhœa, and diseases of the urinary passages, connected with it, or subsequent to it, at the particular request of a very respectable number of gentlemen in London. These

These subjects constituted, formerly, one division of the course of Surgical Lectures delivered every winter; but which his engagements in practice have rendered it inconvenient for him to continue.

We understand, that in addition to an ample detail of the symptoms and treatment of lues venerea, Mr. Pearson enters largely into an account of various appearances resembling those which are produced by the poison of syphilis, but which actually derive their origin from other sources. These are classed under the general term of cachexia syphiloidea, which includes under its subdivisions:—

1st. Diseases appearing after the cure of lues venerea.

2d. Diseases produced by the direct and indirect agency of mercury.

3d. Spontaneous appearances unconnected with syphilis, or the action of the specific remedy.

The history of the cachexia syphiloidea constitutes a very important addition to what has been already collected on the subject of syphilis. And Mr. Pearson thinks himself authorized in asserting, that the symptoms attending this peculiar form of disease are neither less in number, nor less injurious to the human constitution, than those which are derived from the lues venerea.

---

Mr. Parkinson's third volume of the Organic Remains of a former World, will be published in November.

The History of the Royal Society, by Dr. Thomas Thomson, will be published in the ensuing winter, in one volume, quarto, as a companion to the recent Abridgment of the Phil. Trans. The object of this work is to trace the progress of the sciences since the commencement of that illustrious Society, and to take a comparative view of the degree in which they are indebted to British and how much to foreign cultivation. A considerable portion of biography will find a place in this volume.

